# The Mechanical Behavior of Mutant K14-R125P Keratin Bundles and Networks in NEB-1 Keratinocytes

**DOI:** 10.1371/journal.pone.0031320

**Published:** 2012-02-21

**Authors:** Daniel R. Beriault, Oualid Haddad, John V. McCuaig, Zachary J. Robinson, David Russell, E. Birgitte Lane, Douglas S. Fudge

**Affiliations:** 1 Department of Integrative Biology, University of Guelph, Guelph, Canada; 2 Cancer Research United Kingdom (UK) Cell Structure Research Group, College of Life Sciences, University of Dundee, Dundee, Scotland; 3 Institute of Medical Biology, Singapore, Singapore; Dalhousie University, Canada

## Abstract

Epidermolysis bullosa simplex (EBS) is an inherited skin-blistering disease that is caused by dominant mutations in the genes for keratin K5 or K14 proteins. While the link between keratin mutations and keratinocyte fragility in EBS patients is clear, the exact biophysical mechanisms underlying cell fragility are not known. In this study, we tested the hypotheses that mutant K14-R125P filaments and/or networks in human keratinocytes are mechanically defective in their response to large-scale deformations. We found that mutant filaments and networks exhibit no obvious defects when subjected to large uniaxial strains and have no negative effects on the ability of human keratinocytes to survive large strains. We also found that the expression of mutant K14-R125P protein has no effect on the morphology of the F-actin or microtubule networks or their responses to large strains. Disassembly of the F-actin network with Latrunculin A unexpectedly led to a marked decrease in stretch-induced necrosis in both WT and mutant cells. Overall, our results contradict the hypotheses that EBS mutant keratin filaments and/or networks are mechanically defective. We suggest that future studies should test the alternative hypothesis that keratinocytes in EBS cells are fragile because they possess a sparser keratin network.

## Introduction

Epidermolysis bullosa simplex (EBS) is an inherited skin-blistering disease that is characterized by the appearance of fluid-filled blisters after mild mechanical trauma. Blistering arises from rupturing of the keratinocytes of the epidermal *stratum basale* and is most often attributed to dominant genetic mutations in the genes for keratin K5 or K14 proteins [1], [2], [3]. The clinical severity of the EBS phenotype varies from mild to severe and is determined in part by the position of the mutation in the K5 or K14 genes [2], [4], [5]. In severe cases of EBS (known as Dowling-Meara, or EBS-DM), a diagnosis is confirmed by the detection of an intraepidermal cleavage in the *stratum basale* using immunohistochemistry or electron microscopy. In addition, basal keratinocytes within EBS-DM patients typically possess numerous aggregates in the cytoplasm formed by non-filamentous keratin protein [6], [7]. Mutations associated with the EBS-DM type are typically found in the highly conserved boundary regions of the central α-helical rod domains of keratin proteins; these boundary motifs are particularly important in filament assembly [8]. The most commonly altered amino acid residue is the arginine at position 125 of K14, which accounts for 70% of all EBS-DM cases [9].

Genetic studies of EBS patients as well as experiments with transgenic cells and mice [10], [11], [12] provide compelling evidence for a causal link between mutations in K5/K14 genes and EBS, although the exact biophysical mechanism of basal keratinocyte fragility in EBS patients remains unknown. Several hypotheses have been proposed in the literature to explain the mechanical fragility of EBS keratinocytes. The most cited and most plausible of these mechanisms are: 1. the “fragile filaments” hypothesis [13], [14] which posits that K5/K14 filaments formed from EBS mutant proteins are mechanically defective, 2. the “fragile networks” hypothesis [15] which claims that inappropriate interactions among K5/K14 filaments induce mechanical defects of the keratin network in EBS cells, and 3. the “sparse network” hypothesis [12] which claims that the presence of K5/K14 aggregates in EBS cells corresponds to a decrease in the density of the keratin filament network, which is less able to withstand mechanical stress than denser wild-type (WT) networks.

Each of these hypotheses is able to explain various aspects of EBS pathophysiology and experimental data. Our recent work on the mechanical properties of intermediate filaments (IF) (of which keratin filaments are one kind) suggest that these filament networks are remarkably extensible, strong and tough, especially when compared to the other two cytoskeletal elements F-actin and microtubules [16], [17]. These findings are consistent with the fragile filament hypothesis, as disruptive mutations could have serious negative consequences for the material properties of individual keratin filaments. Russell et al. [13] subjected an EBS keratinocyte (KEB-7) cell line to cyclic mechanical stress and found that the keratin network collapses around the nucleus whereas WT networks do not. They also found evidence that the keratin network of these cells fragments into aggregate-like particles when mechanically stressed. On the surface, these results are consistent with the fragile filament hypothesis, but it is also possible that network breakdown was not caused directly by mechanical stress on the filaments, but rather by a generalized cellular stress response that then led to changes to the keratin network.

In vitro investigations of K5/K14 filament suspensions are consistent with the fragile network hypothesis. These studies demonstrate that networks of filaments formed from EBS mutant keratin proteins are less stiff and less resilient than WT networks and appear to be deficient in their ability to form keratin bundles [15]. Furthermore, the fact that EBS-like diseases can be caused by mutations in genes for IF cross-linking proteins like plectin suggest that IF-IF interactions are important for cell integrity and may contribute to the EBS phenotype [18], [19]. The sparse network hypothesis is consistent with observations that keratin filament densities are typically lower in EBS cells, especially those in which keratin proteins are tied up in aggregates [20]. It is also supported by the fact that individuals homozygous for a K14 null mutation and conditional knockout mice lacking K5/K14 filaments both exhibit the EBS phenotype [12], [21], [22], [23].

In the current study, we focused on testing the fragile filament and fragile network hypotheses by subjecting keratinocytes expressing WT and EBS mutant K14-GFP to large scale uniaxial stretches. The GFP tags allowed us to monitor the morphology of the keratin network in live cells during stretch and test the prediction that keratin bundles and/or networks containing EBS mutant protein exhibit a defective response to being loaded in tension. We also investigated the possibility that the presence of mutant keratin proteins in EBS cells interferes with the other two cytoskeletal networks, F-actin and microtubules. We accomplished this by examining the response of the same cells to large-scale stretch in the presence and absence of F-actin and microtubule inhibitors.

Here we demonstrate that K5/K14 filaments and networks containing mutant K14-R125P protein do not appear to be mechanically fragile or defective when subjected to large uniaxial cell strains. While the expression of mutant K14-GFP proteins in keratinocytes induced aggregates similar to those found in EBS-DM cells, we found that the expression of these mutant proteins had no negative effects on keratinocyte viability after large-scale stretch. We also found that the presence of mutant protein had no effect on the response of the F-actin and microtubule networks to large-scale stretches. These experiments provide new clues in the quest to understand the biophysical mechanisms of cell rupture in EBS patients, and they also provide insights into the mechanical behavior of keratinocytes and the keratinocyte cytoskeleton at large-scale cell deformations.

## Results

### Western blots

**Figure 1:**
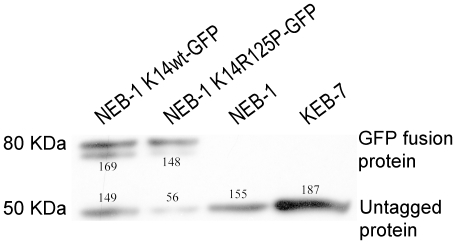
K14 protein expression in NEB-1 K14wt-GFP, NEB-1 K14R125P-GFP, NEB-1 and KEB-7 keratinocytes cell lines. Cell lysates were prepared and equal amounts of protein (30 µg) were detected by Western blotting with the antibody against cytokeratin 14 (LL001). Immunoblot quantification is shown for each band.

To evaluate the expression levels of endogenous and exogenous K14, a Western blot assay was performed on total extracted protein from NEB-1 cells expressing WT keratins, NEB-1 K14wt-GFP cells, NEB-1 K14R125P-GFP mutant cells, and KEB-7 cells, which express one of the most common EBS-DM mutations, K14-R125P. We detected approximately equal amounts of endogenous and exogenous K14 expression in the NEB-1 cells transfected with the K14wt-GFP gene. In the NEB-1 cells transfected with the K14-R125P-GFP, the amount of exogenous K14-R125P-GFP was three times higher than the endogenous K14 protein. Interestingly, both NEB-1 K14wt-GFP cells and NEB-1 K14-R125P-GFP cells displayed similar expression of exogenous K14-GFP and K14-R125P-GFP respectively; however the expression level of endogenous K14 was three times higher in NEB-1 K14wt-GFP cells than the NEB-1 K14-R125P-GFP cells. When we compared the non-transfected NEB-1 cells with the K14-GFP transfected NEB-1 cells, we found that both displayed the same amount of endogenous K14 expression. KEB-7 cells displayed a strong band at 50 KDa, although the ratio of WT K14 and mutant K14-R125P proteins within this band could not be determined (Fig. 1). Most importantly, these results demonstrate that NEB-1 K14-R125P-GFP cells expressed predominantly the mutant form of K14, with WT K14 protein making up only about 30% of the total.

### Osmotic shock induced aggregate production in K14-R125P-GFP expressing cells

**Figure 2:**
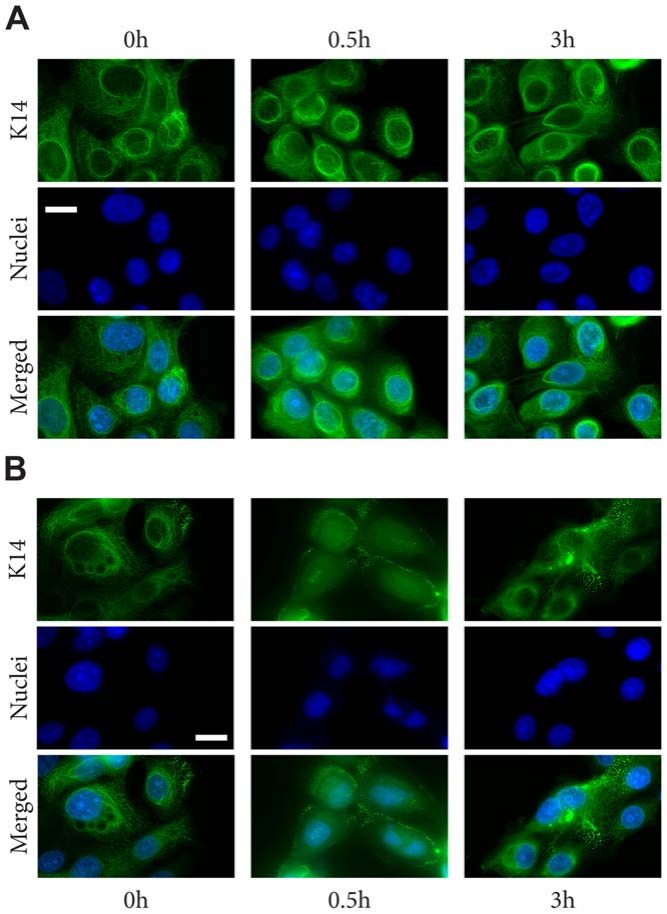
Effect of osmotic shock on the keratin cytoskeleton in the NEB-1 cell line (A) and the EBS derived cell line KEB-7 (B). Both cell lines were subjected to hypo-osmotic shock, then fixed and permeabilized and stained for K14 intermediate filaments. Clear peripheral aggregates were seen in KEB-7 cells 30 min after osmotic shock. No filament fragmentation or aggregates were observed for NEB-1 cells. Scale bar = 22 µm.

**Figure 3:**
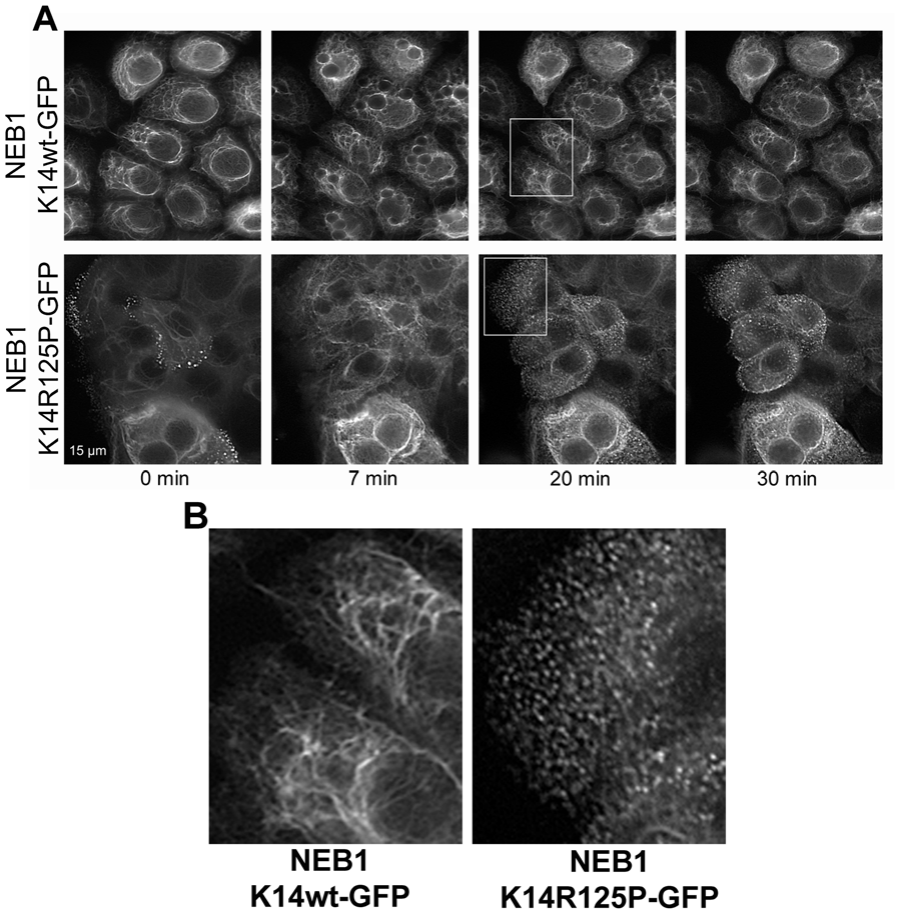
Effect of osmotic shock on the keratin cytoskeleton in the two cell lines studied. A. NEB-1 K14wt-GFP (upper panel) and NEB-1 K14R125P-GFP (lower panel). Both cell lines were subjected to hypo-osmotic shock. Clear peripheral aggregates were seen in R125P cells 20 and 30 min after osmotic shock. No filament fragmentation or aggregates were observed for NEB-1 K14wt-GFP cells. Scale bar = 15 µm. B. Insets from Figure 3A, 20 min after osmotic shock, showing K14 aggregates in the NEB-1 K14R125P-GFP cells, and reconstituted K14 filaments in the NEB-1 K14wt-GFP cells.

The overall goal of this study was to investigate the behavior of WT and EBS mutant keratin bundles and networks in live cells. For this reason, cells were handled in a way that minimized stressors that might induce the appearance of aggregates in mutant cells. However, to confirm that our mutant cells behaved like other EBS cell models, we exposed them in separate trials to an osmotic shock protocol that has been shown to induce aggregate production in EBS mutant keratinocyte lines [4] (Fig. 2). As expected, mutant cells exhibited numerous small K14-R125P-GFP aggregates throughout the cytoplasm after 20 min of exposure to the urea treatment (Fig. 3). Cells expressing WT K14-GFP exhibited some network disruption caused by the appearance of vacuoles in the cytoplasm, but no aggregates (Fig. 3). Both cell lines recovered their original appearance after 1 h of recovery in media for WT cells and 4 h for mutant cells (data not shown).

### Mutant keratin networks survive extreme mechanical strain

**Figure 4:**
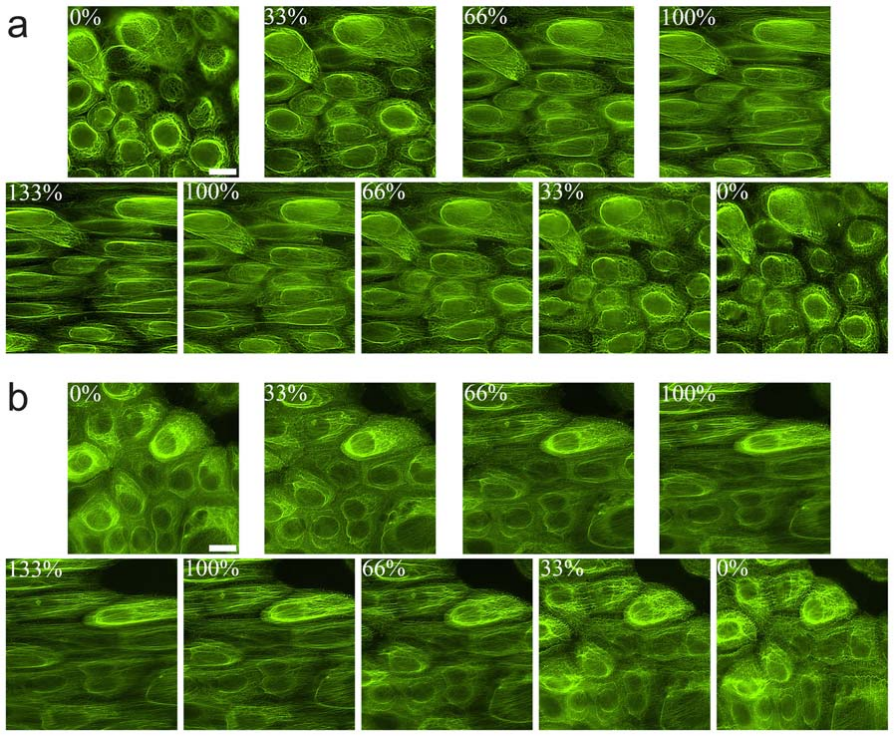
Fluorescent images of (A) NEB-1 K14wt-GFP and (B) NEB-1 K14R125P-GFP keratinocytes undergoing incremental uniaxial strain. The average cell strain is depicted in the top left corner of each image. The cytokeratin networks of the NEB-1 K14R125P-GFP cells withstood extreme cellular strains of 133% and there was no evidence of intermediate filament bundle rupture or the development of keratin aggregates (n = 10). Scale bar = 20 µm.

**Figure 5:**
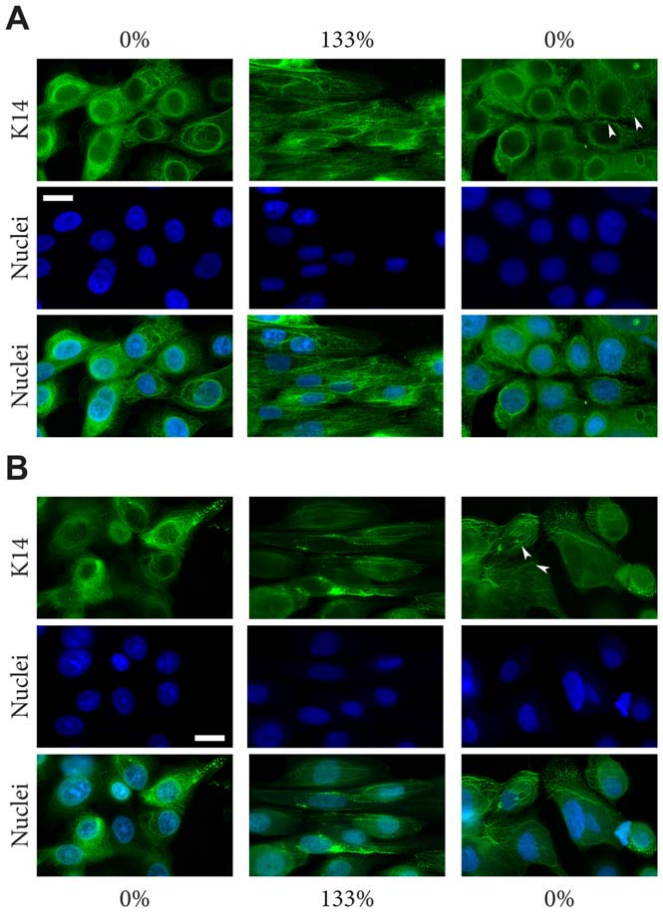
Fluorescent images of (A) WT NEB-1 cells and (B) EBS mutant (R125P) K14 KEB-7 keratinocytes undergoing incremental uniaxial strain. The average cell strain is depicted in the top (A) or the bottom (B) of each panel. Both cell lines were subjected to stretch, then fixed and permeabilized and stained with the anti-K14 (LL001, Santa Cruz) monoclonal antibody. The cytokeratin networks of the EBS cells withstood extreme cellular strains of 133% and there was no evidence of intermediate filament bundle rupture or the development of keratin aggregates. Arrowheads indicate wavy K14 intermediate filaments. Scale bar = 20 µm.

**Figure 6:**
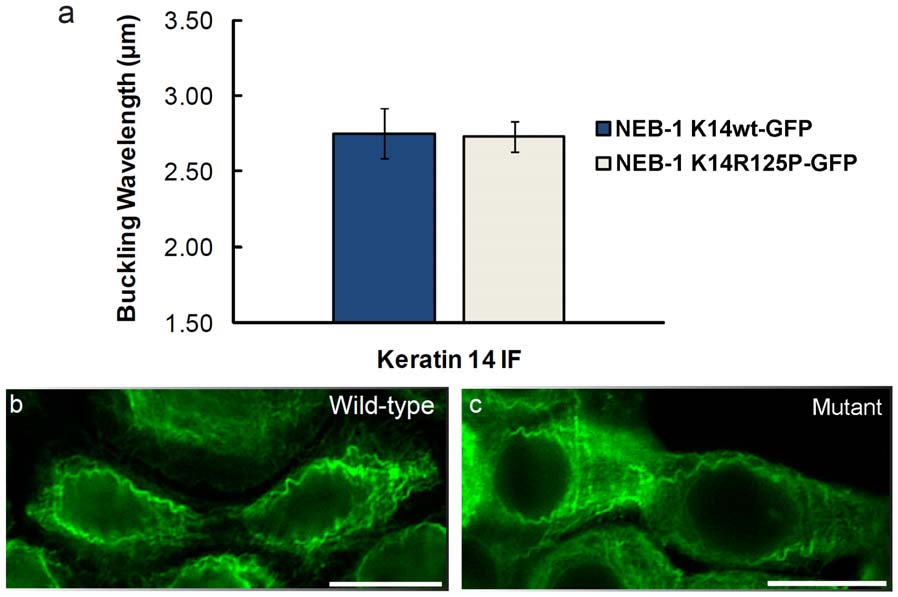
Fluorescent images of the K5/K14 network in keratinocytes after undergoing an extreme strain of 133%. (a) The wavelengths of intermediate filament bundles from both cell lines were not statistically different (p = 0.924). Error bars are standard error. (b) WT and (c) mutant (R125P) K14-GFP networks. Scale bar = 20 µm.

Keratinocytes with WT K14 and mutant K14-R125P networks remained intact and adherent to the silicone membrane before, during and after large-scale uniaxial cell deformations. There were no visible differences between the cell lines before or after stretch with regards to cell shape, appearance, or cell-cell contact using DIC microscopy. The fragile filament and network hypotheses predict that the mutant keratin bundles and networks should fracture at a strain that WT networks can withstand. Fluorescent images of cells undergoing incremental stretch show that the mutant keratin network was able to withstand extreme strains of up to 133% without adverse effects on filament bundles or the filament network and was indistinguishable from the WT controls (Figure 4; Figure S1). To confirm that the GFP tag does not interfere with filament assembly and dynamics, incremental stretch experiments were performed on the WT NEB-1 cells and the EBS-derived KEB-7 keratinocytes (Fig. 5). Immunofluorescence images showed similar behavior of the K14 intermediate filaments and the K14 GFP tagged cell lines (Fig. 5). Large magnitude stretch and release trials showed that keratin bundles of WT and mutant cells displayed a similar inherent waviness along the stretch axis as the strain was decreased (Fig. 6). This waviness has been previously reported in WT keratin bundles [16], [24], and is described as a buckling phenomenon resulting from the compression of filaments embedded in an elastic medium [25], [26]. The buckling wavelength is dependent on the flexural rigidity of the bundle as well as the elastic modulus of the surrounding medium. Wavelengths of buckled filament bundles measured from FITC images were 2.75±0.16 µm (SE) for the WT and 2.73±0.10 µm (SE) for the mutant. The results of the incremental stretch experiment suggest that the extensibility and flexural rigidity of keratin bundles are not altered by the K14-R125P mutation.

### Expression of K14-R125P-GFP has no negative effects on cell viability after stretch

**Figure 7:**
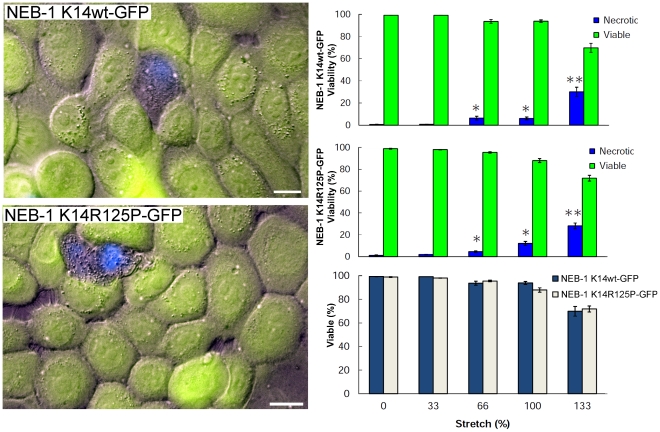
The green vital inclusion dye FDA and blue vital exclusion dye DAPI were used to test for necrosis after extreme strain in NEB-1 K14wt-GFP and NEB-1 K14R125P-GFP cells. The keratinocytes were stretched to a specific strain and then returned to the relaxed state for viability staining. Necrosis increased significantly with increasing cell strain (* = p<0.05; ** = p<0.001). No significant difference in viability was found between the NEB-1 K14wt-GFP and NEB-1 K14R125P-GFP keratinocytes after undergoing extreme uniaxial strain of 133% (p>0.1). Error bars are standard error. Scale bar = 20 µm.

To test whether EBS-DM cells exhibit greater strain-induced necrosis compared to WT cells, we measured cell viability using FDA and DAPI staining immediately after the stretch treatment. Our results showed no significant difference (p>0.1) in viability between the WT and mutant cell lines at extreme cellular strain (133%) with necrosis values of 30.2±4.1% (SE) and 28.2±2.6% (SE), respectively (Fig. 7).

### Response of the F-actin and microtubule networks to cellular stretch

**Figure 8:**
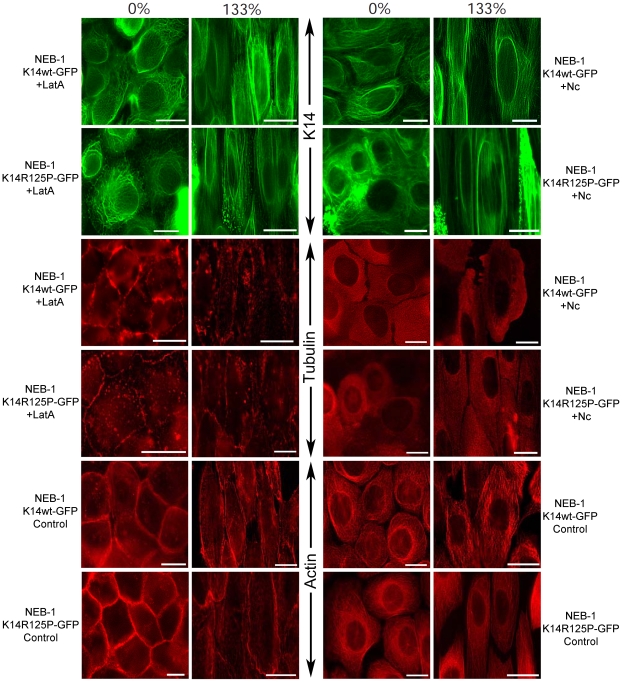
Fluorescent images of the K5/K14, F-actin and microtubule networks in NEB-1 K14wt-GFP and NEB-1 K14R125P-GFP keratinocytes fixed at 0% or 133% strain. Cells were treated with 1 µg/mL nocodazole (Nc) to disturb the F-actin network, or were untreated (control). The F-actin network was visualized with rhodamine-phalloidin (100 nM), and α-tubulin with immunofluorescence. K14-GFP proteins were expressed and visualized by fluorescence microscopy. Scale bar = 20 µm.

**Figure 9:**
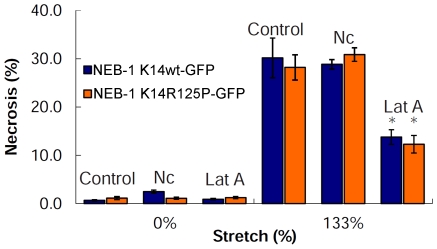
The viability of NEB-1 K14wt-GFP and NEB-1 K14R125P-GFP keratinocytes before (0%) and after (133%) extreme uniaxial strain. The treatments consisted of 1 µg/mL nocodazole (Nc) to disrupt the microtubule network and 0.5 µM latrunculin A (Lat A) to disrupt the F-actin network (n = 6). There was no significant difference in viability between the control cells and cells treated with nocodazole at extreme strain (p>0.1). Cells treated with Lat A exhibited significantly lower necrosis in both cell lines compared to the control (p<0.01).

There were no visible differences in the morphology of the F-actin and microtubule networks between WT and mutant cells at 0% or 133% strain (Fig. 8). Cortical F-actin appeared to thin out as stretch increased, whereas the microtubule network appeared to actively remodel and became more aligned with the stretch axis as the cells were strained. Disruption of the F-actin and microtubule networks had no obvious effect on the morphology of the keratin network in both cell lines, nor was the response of the keratin network to strain affected (Fig. 8). These results suggest that the structural integrity of the keratin network in well-adhered, mostly confluent keratinocytes is not dependent on F-actin or microtubule networks. Disruption of F-actin and microtubules also had no negative effects on cell viability in both cell lines after 133% strain (Fig. 9). In fact, disrupting F-actin significantly decreased stretch-induced cellular necrosis by half in both cells lines (p<0.01), with values of 13.8±1.5% (SE) for WT and 12.3±1.8% (SE) for mutant cells. In contrast, disruption of microtubules had no significant effect on the viability of mutant or WT cells at extreme strain, with nocodazole-treated cultures exhibiting necrosis values of 28.8±1.0% and 30.9±1.4% respectively (Fig. 9).

## Discussion

The results of our uniaxial stretching trials suggest that filaments and networks formed from mutant K14-R125P proteins do not differ from WT filaments and networks in their response to large-scale uniaxial cell deformations. Specifically, we observed no evidence of keratin bundle or network rupture when the cells were subjected to uniaxial strains as high as 133%. These results undermine both the fragile filament and fragile network hypotheses described in the Introduction, although it is possible that even higher levels of uniaxial stretching could reveal differences between WT and mutant networks. It is important to note that in these experiments we were not able quantify the stress developed by the keratin filaments or network, only their morphological response to being deformed. To confirm the idea that mutant filaments do not differ in their material properties from WT filaments will require the application of emerging atomic force microscopy methods developed for the mechanical testing of single intermediate filaments [27], [28].

A study by Russell et al., (2004) [13] is the only other (aside from this one) to investigate the effects of mechanical stress on the morphology of WT and EBS mutant networks in keratinocytes. They subjected cells to a radial oscillatory strain regime (4 Hz at 12% strain), which resulted in breakdown of the keratin network and the appearance of aggregates in EBS mutant cells in as little as 15 min after initiation of the oscillating stretch regime. Taken together with our findings, these data suggest that mutant EBS cells respond differently to deformations that are fast, oscillating, and radial, versus those that are slow, acute and uniaxial. The differences in the results from our study and Russell et al. [13] could be explained by two distinct mechanisms. One possibility is that mutant filaments are identical in their tensile mechanical behavior to WT filaments when they are loaded in a quasi-static (i.e. slow) mode to high strains, but they fall apart when they are loaded repeatedly to low strains at high strain rates. Another possibility is that the filaments that form in EBS mutant cells are mechanically identical to those in WT cells, but fast mechanical oscillations in mutant cells lead to a generalized cellular stress response that then leads to a breakdown of the keratin network via active cellular signaling mechanisms.

The lack of evidence for a disruptive effect of the R125P mutation on the morphology of the keratin network in keratinocytes contradicts results by Ma et al. (2001) [15], who report significant mechanical differences between gels formed from WT K14 and K14-R125C proteins in vitro. The authors attribute these differences to a bundling defect in mutant filaments. Observations of GFP labeled WT and mutant networks in live keratinocytes revealed no such bundling defect, although it is possible that defects may yet be revealed by electron microscopy.

In a previous study, we demonstrated that NEB-1 cells expressing WT K14-GFP are able to survive dramatic uniaxial strains [16]. In the current study, we found that cells expressing the dominant negative R125P mutation of the K14 gene associated with severe EBS are just as capable of surviving large-scale uniaxial strains (Fig. 6). These results raise the important question of whether EBS mutant cells with intact keratin networks are as strong as WT cells. We showed that the two cell lines are capable of surviving the same magnitude of strain (i.e. have similar extensibility), but what if cell viability were measured as a function of stress (i.e. load per area)? Would the mutant cells be able to bear loads of the same magnitude as WT cells before failing? A recent study of EBS mutant and WT keratinocyte mechanics by Lulevich et al. (2010) [11] provides some clues. In this study, the researchers measured the mechanical response of cultured cells (both primary and immortalized) to mechanical compression using an atomic force microscope. They found that not only are keratinocytes far stiffer and more resilient than other cell types, they also found that KEB-7 cells (bearing the K14-R125P mutation) are more compliant and weaker than WT cells. Comparing the results of this study to ours is challenging because of the difference in mechanical loading (apical compression vs. lateral tension). Clearly future studies in which cell strength is measured as a function of network and aggregate density will yield deep insights into the mechanical basis of cell fragility in EBS.

We found that expression of K14-R125P proteins had no obvious effects on the morphology of the microtubule network in keratinocytes either before or during large-scale stretches. We also found that disruption of the microtubule network with nocodazole had no effect on the response of the WT or mutant keratin network or on cell viability after stretch. In contrast to what we saw with the F-actin network, the microtubule network did not appear to be damaged by large-scale stretch. In fact, a comparison of relaxed and stretched cells suggests that the microtubule network was actively remodeled in the 20–30 s that it took to stretch the cells to 133% strain, resulting in microtubules that were more aligned with the stretch axis than mere passive alignment would predict. These observations suggest that further work on the response of the microtubule network to large scale strains could yield new insights into the ways that cells sense and respond to extreme mechanical strains.

Visualization of the F-actin network with rhodamine phalloidin revealed that the K14-R125P mutation did not affect the (mostly cortical) distribution of F-actin in keratinocytes, and nor did it affect the response of the F-actin network to stretch. Although the microtubule network showed evidence of active remodeling as cells were stretched, there was no such evidence for the F-actin network. Cortical actin appeared thinner and in some cases damaged in stretched cells. Interestingly, disruption of the F-actin network with Latrunculin A caused a significant decrease in stretch-induced necrosis. While the precise mechanism underlying this effect is unknown, it is likely related to the higher stresses that develop within cells with intact cortical actin at a given strain than those in which the actin has been disrupted by Latrunculin A.

In summary, we provide evidence that contradicts the idea that cell fragility in EBS is unrelated to the presence of aggregates and caused simply by mechanically defective filaments and/or networks. Future work should focus on testing the sparse network hypothesis and should employ mechanical testing regimes (such as shear testing) in which the rupture strength of the cells can be quantified as a function of keratin network and aggregate density.

## Materials and Methods

### Cell culture

NEB-1 immortalized keratinocytes, expressing WT K5 and K14 [29], were transfected with either WT (NEB-1 K14wt-GFP) or mutant (NEB-1 K14R125P-GFP) constructs in a pEGFP-N1 vector. Transfection was carried out using electroporation and cells were selected using G418 (neomycin). The KEB-7 keratinocyte line was derived from an individual carrying an R125P mutation in the K14 1A domain [25]. All experiments described in this study were carried out on cells between passage 10 and 20 post-immortalization. Cell lines were cultured in 75% DMEM/25% Hams F12 medium, containing 10% fetal calf serum (FCS) and additional growth supplements hydrocortisone (0.4 µg/ml), transferrin (5 µg/ml), lyothyronine (2×10^−11^ M), adenine (1.9×10^−4^ M), insulin (5 µg/ml), and EGF (10 µg/ml). These cell lines are fibroblast feeder cell independent and were cultured at 37°C in 5% CO_2_.

### Osmotic shock

To confirm that cells expressing the mutant R125P version of K14-GFP were capable of producing aggregates like the ones seen in cells cultured from EBS patients, NEB-1 K14wt-GFP and NEB-1 K14R125P-GFP cells were subjected to an osmotic shock treatment that in previous studies was shown to induce network breakdown and aggregate production in mutant cells. Cells were cultured for 48 hours to reach 80% confluence and then subjected to hypo-osmotic shock by immersion in 150 mM urea for 5 min at 37°C [4]. Cells were then returned to normal culture medium and recovery of the K14-GFP network was observed using fluorescence microscopy at 7, 20 and 30 min after the osmotic shock treatment was removed. To confirm that our NEB-1 K14R125P-GFP cells behaved like other EBS cell models, osmotic shock assays were performed on the NEB-1 and the EBS-derived KEB-7 keratinocytes. Cells were fixed with 2% paraformaldehyde at 30 min and 3 h after osmotic shock treatment. Cells were then permeabilized with 0.5% Triton X-100 and stained with an anti K14 monoclonal antibody (LL001, Santa Cruz, sc-53253), and incubated with Alexa fluor 488-conjugated goat anti-mouse IgG (Invitrogen) before fluorescence microscopy observations (Nikon, Eclipse 90i).

### Protein extraction and Western blotting

Cells were washed twice with PBS. Lysis buffer, containing 9 M urea, 2.5 mM EDTA, 2.5 mM EGTA, 1% DTE, 4% CHAPS, was added and left for 45 min at room temperature (vortexing every 10 min). Cells were then harvested and centrifuged at 230,000 g for 90 min at 21°C. Supernatant was then decanted and protein concentration was determined by the Bradford method, and standardized using BSA. Equal amounts of protein (30 µg) (Figure S2) were separated on 12% SDS-PAGE under reducing conditions and transferred to nitrocellulose membranes. Blots were probed with an anti-K14 antibody (LL001, Santo Cruz, sc-53253) and developed with ECL reagents. Protein levels were quantified from images of blots using ImageJ software (NIH Image, Bethesda, USA) and integrating the pixel intensity of each band.

### Cell stretching

Cells were seeded onto 34×14 mm rectangles of collagen IV coated silicone rubber membranes (Flexcell International, USA) and grown to 80–90% confluence. Membranes were clamped individually into the uniaxial cell-stretching device and stretched. The adherent cells on the silicone strip were covered with growth media and 20 mM HEPES buffer (to keep pH stable outside the incubator). To visualize the adhered cells, the stretching device was mounted on a Nikon Eclipse 90i epifluorescence microscope (Nikon Instruments Inc., Canada). Images were captured from a Q-Imaging Retiga EXi cooled monochrome camera using NIS-Elements AR 3.0 software. Captured images were optimized for analysis and publication using the Levels and SmartSharpen adjustment tools in Adobe Photoshop CS4 (Adobe Systems, San Jose, California).

Two kinds of acute cell straining protocols were employed: incremental stretch and stretch-release trials. In incremental stretches, the traveling end of the rubber strip was extended in increments of 40% of the resting length using the micrometer. Cells were stretched at a rate of 0.30 mm s^−1^, which corresponded to a strain rate of 0.013 s^−1^ given that the resting length of clamped membranes was 22 mm. Each increment of stretch took ∼30 s to reach the desired strain. At each strain, a differential interference contrast (DIC) and fluorescence image was taken. When the maximum cell strain of 133% was reached, a similar procedure was followed back to 0% strain. These trials took about 30 min to complete. During the stretch-release trials, cells were strained at a strain rate of 0.013 s^-1^ to the desired amount and immediately allowed to return back to resting length for viability staining (see below) and imaging.

Values of micrometer strain did not correspond to the strain in the rubber substrate or the strain exhibited by the cells, due mainly to unavoidable slippage of the membrane from the clamps and partial loss of cell adhesion to the substrate. The relationship between micrometer strain and cell strain was quantified [16] and, in all figures, the strain reported is cell strain.

### Cell Viability

Viability of 80–90% confluent keratinocytes after uniaxial stretch treatments of 0% (control), 33%, 66%, 100% and 133% was assessed using the vital inclusion dye fluorescein diacetate (FDA) [30] and the vital exclusion dye 4-6-diamidino-2-phenylindole (DAPI) [16]. After stretching at a rate of 0.03 s^−1^, cells were stained for one min using 0.01 mg/mL FDA dissolved in media, followed by three rinses with PBS. Cells were next stained for three s with 3.6 mM DAPI in PBS, followed by three rinses with PBS. The elapsed time the cells were outside the 37°C incubator was 10 min, which included stretching, staining and imaging. Cells that stained with FDA and excluded DAPI were considered viable, whereas cells that excluded FDA and stained with DAPI were considered necrotic. Staining with FDA and DAPI were overwhelmingly mutually exclusive. The very small fraction of cells that stained with neither or both of the stains were excluded from the analysis. Viability data were collected from six independent cell-stretching trials for each treatment, which amounted to approximately 10,000 cells per treatment. One-way ANOVA was performed on arcsin transformed viability percentage data.

### F-actin staining and network disruption

Live keratinocytes were subjected to a control (0%) or stretch (133%) treatment and then fixed with a phosphate buffered 3% paraformaldehyde solution for 10 min at 4°C followed by two rinses of PBS. Permeabilization of the cells was carried out using 0.2% Triton X-100 in PBS for 10 min at 4°C followed by two rinses of PBS. Fixed cells were stained with rhodamine phalloidin (100 nM, Cytoskeleton Inc. Cat# PHDR1) at room temperature for 40 min in the dark. Five washes of PBS were then applied and the F-actin network visualized with a TRITC filter cube on a Nikon epifluorescent microscope.

To disrupt the F-actin network in keratinocytes, latrunculin A (0.5 µM; CalBioChem Cat# 428021) was applied to the culture plates in the incubator for 1 hour. These cells then underwent strains as high as 133% and were fixed and stained for visualization under the epifluorescent microscope. The concentration of latrunculin A used allowed for disruption of the F-actin network without affecting the viability of the cell. Washing out of the latrunculin A resulted in the full recovery of the network after 1 hour.

### α-tubulin staining and microtubule network disruption

To stain for α-tubulin, we used a primary mouse monoclonal antibody against mammalian α-tubulin (Invitrogen Cat# A11126) followed by a goat anti-mouse secondary IgG antibody (Alexa Fluor 555 F(ab′)_2_, Invitrogen Cat# A21425). The live keratinocytes were subjected to a stretch treatment and then fixed with a phosphate buffered paraformaldehyde/glutaraldehyde (3%/0.1%) solution for 10 min at 4°C followed by two rinses of PBS. Cells were permeabilized with 0.2% Triton X-100 in PBS for 10 min at 4°C followed by two rinses of PBS. Incubations of fixed cells with primary (2 µg/mL) and secondary antibodies (10 µg/mL) were both carried out at room temperature for 45 min in the dark and rinsed two times with PBS between each step. The labeled microtubule network was visualized through the TRITC filter on the same Nikon epifluorescence microscope and imaging system.

To disrupt the microtubule network in keratinocytes, nocodazole (1 ug/mL) was added to the culture plates in the incubator for 1 hour. These cells were then subjected to strains as high as 133% and were fixed and stained for visualization under the epifluorescent microscope. The concentration of nocodazole used allowed for the disruption of the microtubule network without affecting the viability of the cell, and removing nocodazole by rinsing the cultured cells resulted in the recovery of the network after 1 hour.

## Supporting Information

Figure S1 **Fluorescent image sequence of EBS mutant NEB-1 K14R125-GFP keratinocytes undergoing incremental uniaxial strain.** The average cell strain is depicted in the top left corner of each frame.
(AVI)

Figure S2 **Coomassie staining for the western blot shown in** **Figure 1** **.**
(TIF)
