# Supplementary figures and images for: The Mechanical Behavior of Mutant K14-R125P Keratin Bundles and Networks in NEB-1 Keratinocytes
Source: PLoS One. 2012 Feb 21;7(2):e31320. doi: 10.1371/journal.pone.0031320 (PMC3283645; doi:10.1371/journal.pone.0031320)

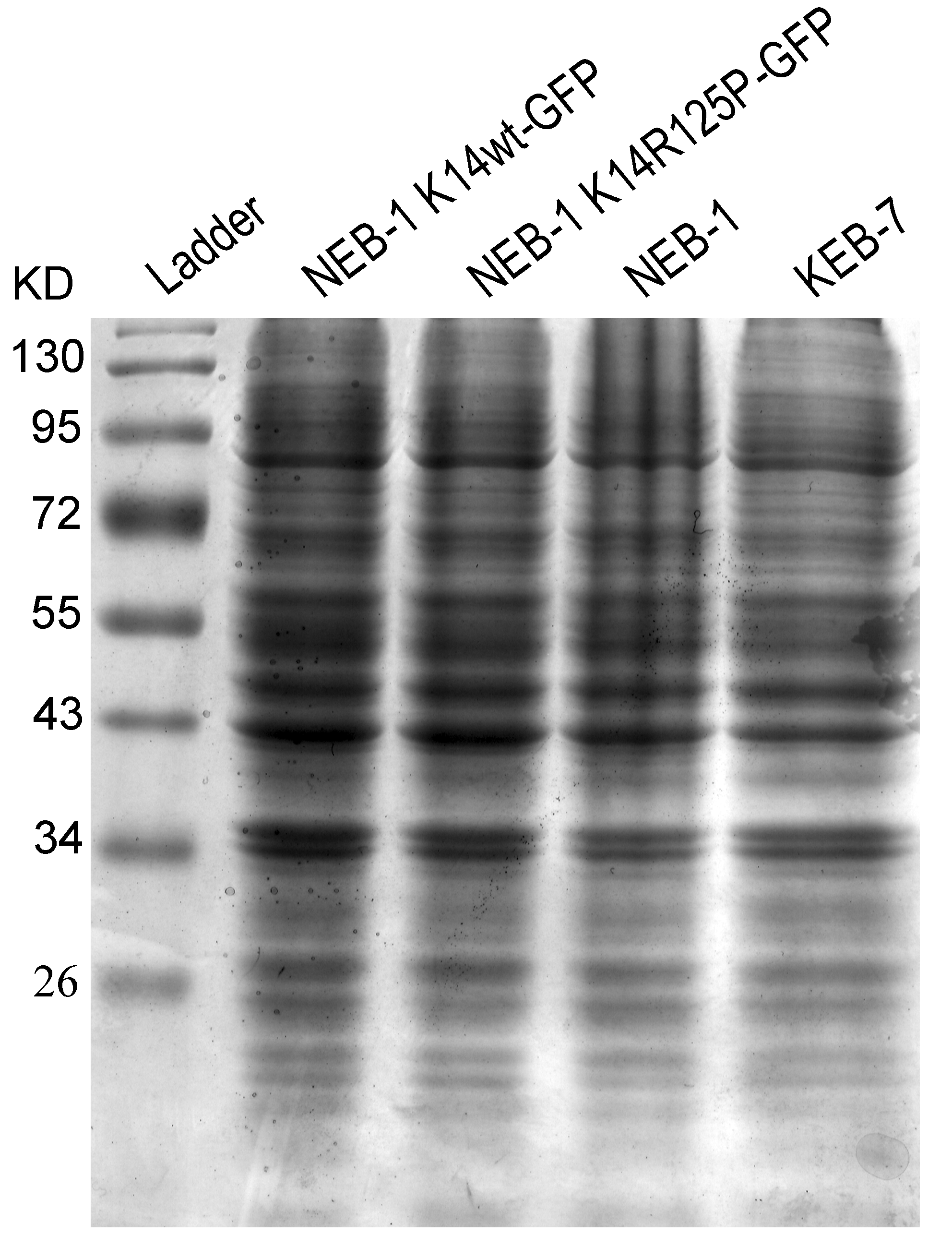

Supplement: Figure S2 — Coomassie staining for the western blot shown in Figure 1 . (TIF) [file pone.0031320.s002.tif]
